# Using gene expression data and network topology to detect substantial pathways, clusters and switches during oxygen deprivation of *Escherichia coli*

**DOI:** 10.1186/1471-2105-8-149

**Published:** 2007-05-08

**Authors:** Gunnar Schramm, Marc Zapatka, Roland Eils, Rainer König

**Affiliations:** 1 Theoretical Bioinformatics, German Cancer Research Center (DKFZ), 69120 Heidelberg, Germany; 2 Department of Bioinformatics and Functional Genomics, Institute of Pharmacy and Molecular Biotechnology, University of Heidelberg, 69120 Heidelberg, Germany

## Abstract

**Background:**

Biochemical investigations over the last decades have elucidated an increasingly complete image of the cellular metabolism. To derive a systems view for the regulation of the metabolism when cells adapt to environmental changes, whole genome gene expression profiles can be analysed. Moreover, utilising a network topology based on gene relationships may facilitate interpreting this vast amount of information, and extracting significant patterns within the networks.

**Results:**

Interpreting expression levels as pixels with grey value intensities and network topology as relationships between pixels, allows for an image-like representation of cellular metabolism. While the topology of a regular image is a lattice grid, biological networks demonstrate scale-free architecture and thus advanced image processing methods such as wavelet transforms cannot directly be applied. In the study reported here, one-dimensional enzyme-enzyme pairs were tracked to reveal sub-graphs of a biological interaction network which showed significant adaptations to a changing environment. As a case study, the response of the hetero-fermentative bacterium *E. coli *to oxygen deprivation was investigated. With our novel method, we detected, as expected, an up-regulation in the pathways of hexose nutrients up-take and metabolism and formate fermentation. Furthermore, our approach revealed a down-regulation in iron processing as well as the up-regulation of the histidine biosynthesis pathway. The latter may reflect an adaptive response of *E. coli *against an increasingly acidic environment due to the excretion of acidic products during anaerobic growth in a batch culture.

**Conclusion:**

Based on microarray expression profiling data of prokaryotic cells exposed to fundamental treatment changes, our novel technique proved to extract system changes for a rather broad spectrum of the biochemical network.

## Background

**Figure 1:**
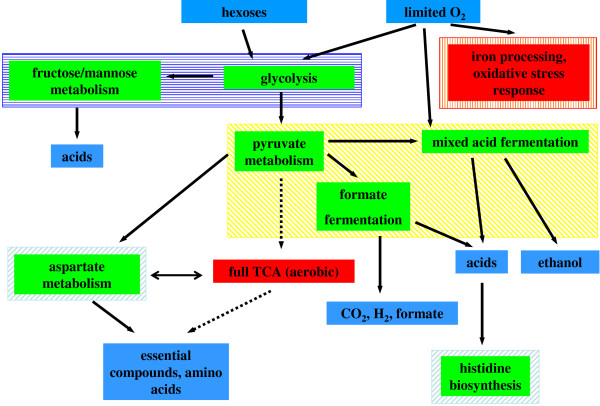
Overview of the found significantly regulated metabolic pathways during oxygen deprivation (green: up-regulated, red: down-regulated, blue: metabolites). For more details, see: Figure 4 (yellow cross-hatched), Figure 5 (blue hatched) and Figure 6 and 7 (red and light blue boxes, respectively). Note that in this Figure, the metabolic pathways of Figure 7 are represented by two boxes. This is due to the unspecific hub-like nature of L-glutamine (see Conclusions).

Over the last decades our understanding of cellular metabolism has increased considerably [1], in particular for less complex organisms such as *Escherichia coli *[2-4]. The gained knowledge includes cellular adaptation programs that respond to changing environmental conditions such as nutrient excess and starvation [5]. Current microarray technology allows for the investigation of all genes of an organism under various conditions, resulting in the generation of a massive amount of expression data. One of the greatest challenge we are faced with is to then analyse the data as a whole and extract the meaningful relationships among specific genes. Standard methods such as SAM [6] or machine learning algorithms [7] are able to detect patterns in gene expression data, distinguishing between different states of a cell. However, the above methods for classification and pattern discovery do not consider interactions between different genes and their corresponding proteins. Functional relationships between genes can be assembled by e.g. regulatory, signal transduction and metabolic networks. Gardner and co-workers used gene expression microarray data to infer a regulatory network for *E. coli *[8]. They developed a linear model and effectively reduced the number of parameters by assuming a sparse regulatory network. Finally, they verified their inferred regulatory network on a smaller subset, i.e. the regulation of the SOS pathway. In a recent study, a large compendium of gene expression microarray data for *E. coli *was analysed using an information theoretical approach revealing new regulatory interactions [9]. When analysing a metabolic network, every enzyme can be represented by its corresponding gene. For sets of genes, pathway scores have been calculated improving the sensitivity to detect crucial enzymatic pathways when taking network distances for enzyme pairs into account [10]. Transcription data and the topological information derived from the metabolic network was connected by calculating Z-scores of highly correlated sub-networks [11]. Genes with common biological processes or functions were grouped by their gene ontology terms [12] and gene set enrichment tests performed on these groupings [13]. Additionally, gene set enrichments were tested by their common pathways in the corresponding networks [14,15]. However, these approaches do not take into account direct interactions within the network. In contrast, a Potts-spin clustering algorithm on metabolic networks was developed depending on direct nearest-neighbour relationships. It was applied yielding sub-graphs stimulated by environmental conditions [16]. Furthermore, common gene expression levels of neighbouring nodes in a metabolic network were calculated by averaging over all neighbours of a gene and revealed several interesting regulated pathways for the human immune system [17]. Rapaport and co-workers extracted gene expression patterns of neighbouring genes in the network yielding good classification of the profiled samples by calculating Fourier transformations and rejecting high frequency signals [18]. However, these approaches did not consider switch like behaviours of neighbouring genes. To detect common *and *contrasting tendencies, an image-like representation of the cellular metabolism can be used by interpreting expression levels as pixel intensities with grey values and the network topology as relationships between pixels. Image processing methods may then be applied to extract crucial features from such an image. Wavelet transforms are such an image processing method and were applied for texture classification [19], for feature generation to automatically classify microscope images [20] and large-scale functional genetic screens [21]. Without taking any network information into account, wavelet transforms have been used together with other image processing methods for analysing microarray data [22,23], in particular the Haar wavelet power spectrum for feature selection [24]. The application potential of this powerful technology to analyse biological networks is clear, yet challenging. While the underlying topology of an ordinary image consists of a lattice grid, biological networks have a rather scale-free architecture [25]. We recently reported one approach that applied image processing methods on the two-dimensional and therefore image-like adjacency matrix of the network [26]. In the present study we expand upon this method using the original architecture of the metabolic network. We analysed gene expression changes for each pair of neighbouring nodes combining their values additive (common response) and subtractive (opposing response, switch like behaviour). In a second step all combined nodes with a common response were again combined to yield significant clusters of co-expression. Such a simple approach allowed the analysis of the cellular stress response, not only for highly connected regions of the network but also for linear chains as well as the identification of specific switches. We analysed gene expression changes of *E. coli *during oxygen deprivation. With this technique we were able to detect the expected substantial regulatory adaptation programs, including up-regulated formate fermentation, mixed acid fermentation, metabolisms of hexoses and down regulation of the respiratory TCA cycle (see Figure 1). Furthermore, our technique revealed a down-regulation of the iron processing metabolism due to reduced oxidative stress during oxygen deprivation. The revealed up-regulation of the histidine biosynthesis pathway may constitute the adaptive response of *E. coli *to an acidic environment due to the excretion of acidic products during anaerobic growth in a batch culture.

## Results and Discussion

### Testing the method with simulated data

#### Setting up the network and calculating the simulated expression data

**Figure 2:**
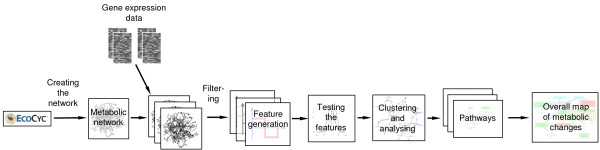
General workflow of the method. The metabolic network of *E. coli *was put up using the EcoCyc database. Gene expression data was mapped onto the reactions of the network resulting in an image like representation (red boxes). Features were generated by using the Haar wavelet transformation on every connected reaction pair. The most discriminative features were identified by a t-test. Sub-graphs were built by connecting significant reaction pairs. Regions with identical regulation of more than four reactions were extracted (clusters). Reaction pairs with opposite regulation were identified as switches and were also extracted. The resulting pathways were analysed by literature scanning in-depth. Assembling the found pathways yielded an overall picture of the metabolic processes.

To test our method with simulated data on a simplified model network, we constructed a regular grid of 30 × 40 artificial reactions (workflow see Figure 2). On this simulated image-like metabolic network we randomly selected pathways of connected reactions with lengths 7, 10 and 24. These lengths corresponded to an expected length of a biological pathway (7, 10) and to the most frequent path length of the shortest paths between all pairs of nodes in the regular grid, respectively. 100 runs were performed generating 44 experiments of simulated expression data with a ground level of 6, in rather good agreement with our normalised gene expression data. To this, a Gaussian noise of mean 0 and standard deviation 1 was added. Two classes were formed with 22 experiments each. In one class the reactions of the randomly chosen pathways were up-regulated by adding a constant level Δ to the random expression levels.

#### Performing the method on random data

In each run the expression data of all 44 samples (22 class 1 and 22 class 2) were mapped on the nodes (reactions) of the simulated metabolic network. Features were generated by applying the one dimensional Haar-wavelet transform onto each pair of neighbouring nodes. This yielded 9320 features for every sample. A t-test was applied for every feature to rank the features with respect to their discriminating property while correcting p-values for multiple testing (Bonferroni) [27]. Every feature for all reaction-pairs was ranked according to its p-value. The p-value cut-off was set to 0.01. Reactions were regarded as up-regulated if the corresponding simulated genes were significantly differentially expressed (p-value of a t-test ≤ 0.05) and not significantly differentially expressed otherwise. Not differentially expressed end-nodes were discarded. We compared our technique to a standard method.

#### Comparison to a standard method

**Figure 3:**
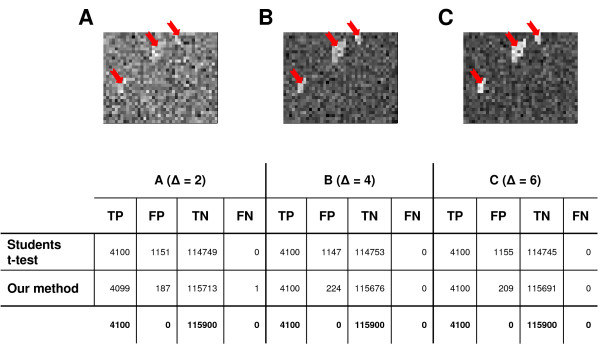
Validation of the method on a regular grid consisting of 40 × 30 reactions (pixel). Random gene expression data was generated and mapped onto the nodes of the grid. The 44 samples were divided into two classes differing only significantly in the reactions of three randomly chosen pathways (red arrows). Up-regulation of these reactions in one class was achieved by adding a constant value Δ to their expression levels. Our technique revealed significantly less false positives (FP) than the standard t-test for all chosen values of Δ. The last row shows the desired outcome after 100 runs (TP: true positives, FP: false positives, TN: true positives, FN: false negatives).

A standard Students t-test was applied on the simulated expression data without taking any network information into account. For both methods true positives, false positives, false negatives and true negatives were calculated. To investigate a broader spectrum for the precision and sensitivity of our technique, the validation was performed with a variety of added constants (Δ = 2, 4, 6). Our technique decreased the number of false positives significantly (Figure 3). In a step further we investigated how our technique performed on a biological network, choosing the metabolic network of *E. coli*, constructed as described in Methods. Out of this network we selected randomly pathways of lengths 5, 7, 10 and performed the same method as described above for different constants (Δ = 2, 4, 6). We obtained a similar superior performance of our approach. The number of false positives was reduced nearly threefold, while the detection power of true positives was identical (Results not shown).

### The metabolism of *E. coli *under oxygen deprivation

The general workflow was briefly as follows:

- Establishing the metabolic network using the EcoCyc database,

- Mapping gene expression data onto the nodes of the network,

- Generating feature modules using the Haar wavelet transformations,

- Statistical testing of the feature modules,

- Clustering of significant reaction pairs,

- Analysing found clusters and switches in depth, and

- Fusing of the results to receive an overall map of metabolic changes.

The expression data of all 43 samples (21 aerobic and 22 anaerobic) from the study of Covert et al. [28] was mapped onto the reactions of the metabolic network. Features were generated as described above using the Haar-wavelet transform yielding 6890 features for each sample. Discriminating features were identified via ranking of p-values from a t-test. Calculated p-values were corrected with a multiple t-test correction for possible mutant influences (see Methods) and for multiple testing (Bonferroni) [27]. The p-value cut-off was again set to 0.01 resulting in 660 significantly discriminating features. All significant reaction-pairs were extracted and connected yielding sub-graphs. In total, five such connected sub-graphs were identified consisting of 165 reactions. Reactions were regarded as up-regulated if the corresponding genes were significantly over-expressed under anaerobic conditions (p-value ≤ 0.05 of a mutant corrected t-test, see Methods), as down-regulated if significantly under-expressed and not significantly differentially expressed otherwise. Neighbouring, connected nodes that showed identical regulation (up or down) were grouped together to simplify interpretation. We refer to these groups as "clusters" in the following. Not differentially expressed end-nodes in a cluster were discarded. The resulting 10 clusters containing at least five reactions were interpreted in more detail and grouped according to their functional role (Table 1, supplement 1 contains the corresponding EcoCyc reaction-ids). Furthermore, pairs of reactions with significantly opposing regulatory behaviour were defined as switches. All significant switches were extracted (p-value ≤ 0.01). 64 such switches were identified. The first 20 switches are discussed in detail (Table 2 shows the first 20 switches, supplement 2 provides all 64 switches). An overview of the extracted pathways is given in the next paragraph.

**Table 1:** Extracted network clusters.

**Pyruvate processing, formate fermentation, anaerobic respiration and anaerobic synthesis of deoxyribonucleosides**	
**1^st ^cluster**	**2^nd ^cluster**
argininosuccinate lyase, aspartate ammonia-lyase, dimethyl sulfoxide reductase, 3,4-dihydroxy-2-butanone 4-phosphate synthase, formate hydrogenlyase complex, formate dehydrogenase pyruvate formate-lyase, fumarate reductase, FocA formate FNT transporter	pyruvate formate-lyase activating enzyme, coproporphyrinogen III oxidase, anaerobic, anaerobic nucleoside-triphosphate reductase activating system, PFL-deactivase, ribonucleoside triphosphate reductase activase, lipoate synthase

**Processing of hexoses**	

**1^st ^cluster**	**2^nd ^cluster**
1-phosphofructokinase, 6-phosphofructokinase, 6-phospho-β-glucosidase, glucokinase, mannitol-1-phosphate 5-dehydrogenase, mannose-6-phosphate isomerase, phosphoglucose isomerase, EIIMan transporter	glyceraldehyde 3-phosphate dehydrogenase, 2-keto-3-deoxy-6-phosphogluconate aldolase, phosphogluconate dehydratase, phosphoglycerate kinase, triose phosphate isomerase

**Iron processing**	

**1^st ^cluster**	**2^nd ^cluster**
2,3-dihydroxybenzoate-AMP ligase, 2,3-dihydro-2,3-dihydroxybenzoate dehydrogenase, serine activating enzyme, aryl carrier protein, enterobactin synthase multienzyme complex, isochorismatase, isochorismate synthase, enterochelin esterase	cysteine desulfurase, selenocysteine lyase, thiamin (thiazole moiety) biosynthesis protein, YaaJ alanine AGSS transporter, valine-pyruvate aminotransferase

**Acid response**	

aspartate-ammonia ligase, asparagine synthetase B, ATP phosphoribosyltransferase, CDP-diglyceride synthetase, CTP synthetase, imidazole glycerol phosphate synthase, histidinal dehydrogenase, histidinol-phosphate aminotransferase, phosphoribosyl-AMP cyclohydrolase, histidinol-phosphatase, histidinol dehydrogenase, phosphoribosyl-ATP pyrophosphatase, imidazoleglycerol-phosphate dehydratase, L-aspartate oxidase, phosphoribosylformimino-5-amino-1-phosphoribosyl-4-imidazole carboxamide isomerase, quinolinate synthase complex, protein-(glutamine-N5) methyltransferase, aspartate DAACS transporter	

**Nucleoside metabolism**	

**1^st ^cluster**	**2^nd ^cluster**
dGDP kinase, nucleoside diphosphate kinase ribonucleoside-diphosphate reductase, deoxyguanylate kinase, GTP cyclohydrolase I, guanylate kinase, guanosine-3',5'-bis(diphosphate) 3'-diphosphatase, ribonucleoside-diphosphate reductase	dTDP-glucose pyrophosphorylase, dTDP kinase, UDP-glucose-hexose-1-phosphate uridylyltransferase, UDP-galactopyranose mutase, nucleoside diphosphate kinase, galactose-1-phosphate uridylyltransferase

**One carbon units**	

gcv system, glycine dehydrogenase (decarboxylating), aminomethyltransferase	glutathione synthetase, glycyl-tRNA synthetase, lipoyl-protein ligase A

**Table 2:** Extracted switches in the network. Significantly differentially expressed pairs of reactions (p-value ≤ 0.01). The first 20 switches are shown here and described in detail in the text.

**Rank**	**Up-regulated reactions**	**Metabolites**	**Down-regulated reactions**	**P-value**
1	formate hydrogenlyase complex	formate	formyltetrahydrofolate deformylase	4.67E-14
2	acetaldehyde dehydrogenase	acetaldehyde	ethanolamine ammonia-lyase	1.25E-12
3	FocA formate FNT transporter	formate	formyltetrahydrofolate deformylase	5.81E-12
4	formate hydrogenlyase complex	formate	GTP cyclohydrolase I	1.65E-11
5	3-methyl-2-oxobutanoate hydroxymethyltransferase	2-dehydropantoate	2-dehydropantoate reductase	2.33E-11
6	serine hydroxymethyltransferase	tetrahydrofolate, 5,10-methylene-THF, glycine	gcv system	6.69E-09
7	serine hydroxymethyltransferase	glycine	glycine dehydrogenase (decarboxylating)	2.07E-08
8	formate dehydrogenase	formate	formyltetrahydrofolate deformylase	1.30E-07
9	2-keto-4-hydroxyglutarate aldolase	glyoxylate	glyoxylate reductase B,glyoxylate reductase	1.74E-07
10	fumarate reductase	fumarate	5'-phosphoribosyl-4-(N-succinocarboxamide)-5-aminoimidazole lyase	2.00E-07
11	fumarate reductase	fumarate	adenylosuccinate lyase	2.00E-07
12	CTP synthetase	UTP	galactose-1-phosphate uridylyltransferase	3.16E-07
13	gluconokinase	gluconate	2-ketoaldonate reductase	1.36E-05
14	phosphoenolpyruvate carboxylase	oxaloacetate	aspartate transaminase	2.00E-05
15	FocA formate FNT transporter	formate	GTP cyclohydrolase I	3.35E-05
16	BrnQ branched chain amino acid LIVCS transporters	L-isoleucine	branched chain amino acids ABC transporters	3.78E-05
17	BrnQ branched chain amino acid LIVCS transporters	L-leucine	branched chain amino acids ABC transporters	3.78E-05
18	BrnQ branched chain amino acid LIVCS transporters	L-valine	branched chain amino acids ABC transporters	3.78E-05
19	3-hydroxy acid dehydrogenase	L-serine	phosphoserine phosphatase	4.33E-05
20	phosphatidylglycerophosphate synthase	a CDP-diacylglycerol, CMP	CDP-diacylglycerol pyrophosphatase	4.78E-05

### Main functional findings

**Figure 4:**
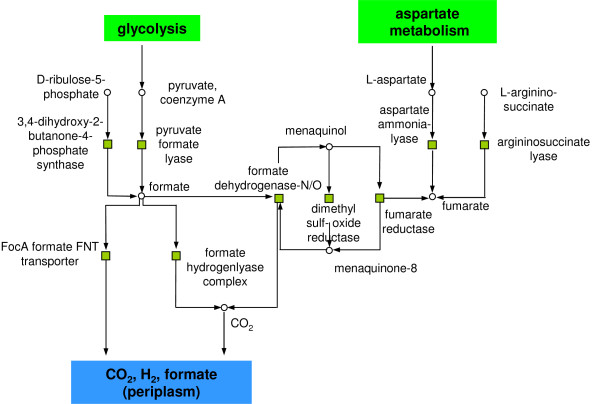
Fermentation of formate was up-regulated processing pyruvate into formate via pyruvate lyase. Pyruvate is degraded to formic acid (formate), which then is either expelled (via transporters), or further degraded into H_2 _and CO_2 _by the formate hydrogenlyase complex (for more details see text). Reactions are symbolised by squares, metabolites by circles. Green (red) squares indicate significant up (down)-regulation (p-value ≤ 0.05) under anaerobic conditions.

**Figure 5:**
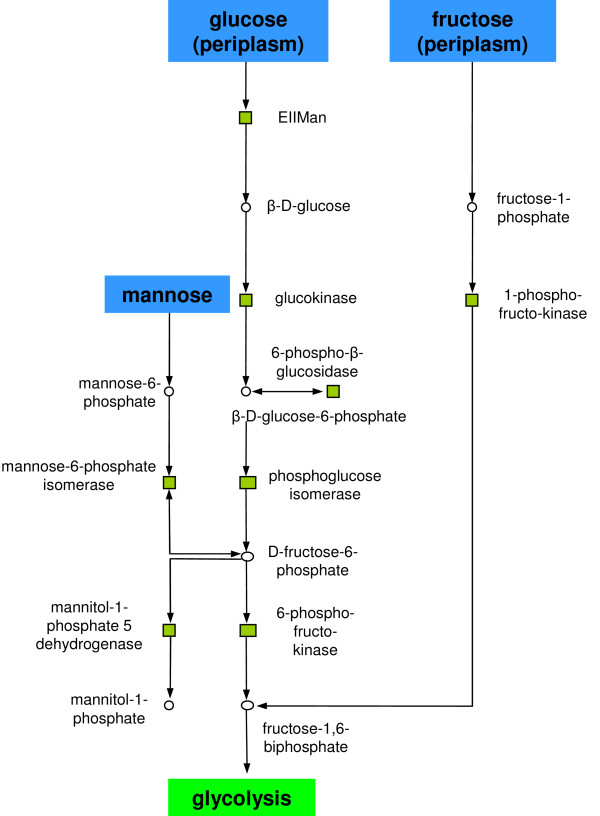
Metabolisms of hexose nutrients under anaerobic conditions. Fructose and mannose metabolism were up-regulated indicating a higher glucose processing. For box colours see Figure 4.

**Figure 6:**
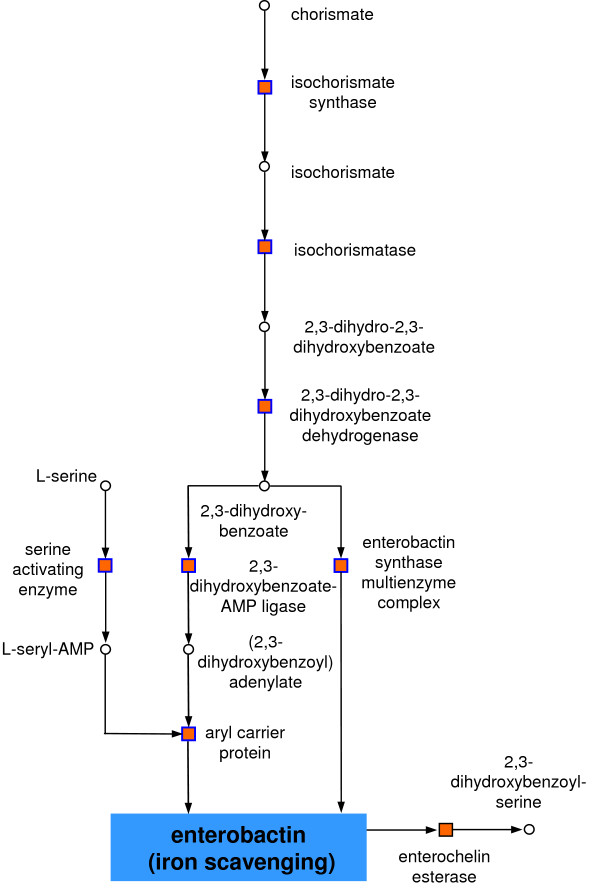
Iron processing in an anaerobic environment. Iron is scavenged by *E. coli *using enterobactin, whose biosynthesis (blue bordered nodes) was down-regulated. For box colours see Figure 4.

**Figure 7:**
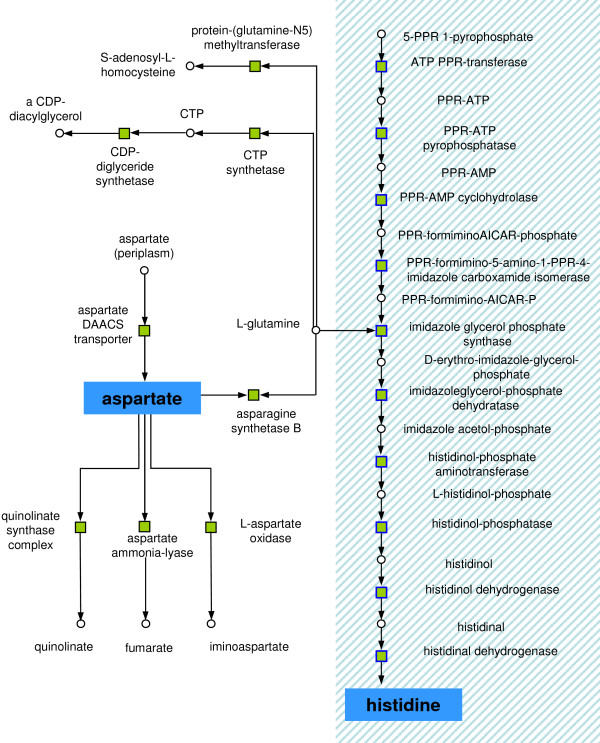
During anaerobic growth *E. coli *performed mixed acid fermentation, resulting in a more acidic environment. The histidine biosynthesis (blue bordered nodes and light blue box) was up-regulated for buffering (see text). For box colours see Figure 4. PPR: phosphoribosyl.

The metabolic network of *E. coli *underwent substantial changes in regulation, when adapting to the environmental change from oxygen rich to deprived conditions (Figure 1). Due to limited oxygen, glycolysis and the fructose/mannose metabolism was up-regulated securing energy production under anaerobic conditions (Figure 5). Furthermore, pyruvate metabolism, formate fermentation and mixed acid fermentation were also up-regulated fermenting the products of the glycolysis (Figure 4). In contrast, iron processing and oxidative stress responses were down-regulated as oxidative stress was reduced (Figure 6). As expected, the aerobic part of the TCA-cycle was down-regulated. The need to generate essential compounds and amino acids was indicated by an elevated level of the aspartate metabolism (Figure 7). An indirect effect of the oxygen rich to oxygen deprived conditions was the up-regulation of the histidine biosynthesis (Figure 7). Histidine may function as a buffer for produced acids accumulating in the batch culture. In the following, these findings are described in detail.

### Functional description of the extracted clusters

#### Pyruvate processing, formate fermentation, anaerobic respiration and anaerobic synthesis of deoxyribonucleosides

Two clusters belonged to this sub-group (Table 1). The first cluster (Figure 4) consisted of nine reactions. Edges were due to the metabolites formate, fumarate and reduced menaquinone. Reactions connected via formate: Pyruvate formate lyase was up-regulated under anaerobic conditions to process pyruvate into formate (fermentation) [29]. Formate degradation into CO_2 _and H_2 _was supported by up-regulated formate hydrogen lyase [30,31] and formate dehydrogenase. Formate release into the periplasm was facilitated by up-regulation of the corresponding transporter (EcoCyc-id: TRANS-RXN-1). Also up-regulated was 3,4-dihydroxy-2-butanone 4-phosphate synthase which functions as the first and rate limiting step in flavin mononucleotide (FMN) biosynthesis. It is notable that FMN functions as an electron mediator during anaerobic respiration [32]. Reactions connected by reduced menaquinone: Dimethyl sulfoxide reductase was up-regulated as it is needed in the anaerobic electron transport chain [33]. Also up-regulated was fumarate reductase which is used by *E. coli *during anaerobic growth [34]. In this reaction menaquinol acts as an electron acceptor, while fumarate can function as a terminal electron donor [35]. Further reactions in this cluster were connected with fumarate reductase via fumarate. Aspartate ammonia-lyase was up-regulated, to process aspartate into fumarate during anaerobic growth on glucose [36,37]. The second cluster contained six up-regulated reactions which were connected by the metabolite *S*-adenosyl-L-methionine. Pyruvate formate-lyase (PFL)-deactivase was up-regulated. This enzyme is a catalyser for quenching and inactivating pyruvate formate-lyase and is expressed under anaerobic conditions to the same levels as pyruvate formate-lyase [38]. Corresponding to this, pyruvate formate-lyase activase was also up-regulated as it activates pyruvate formate-lyase in an anaerobic environment [39]. Pyruvate dehydrogenase requires the lipoate modification of complex subunits [40]. Lipoate synthase is needed for the biosynthesis of lipoic acid and is necessary for the anaerobic glycine cleavage system activity [41]. Note that reactions belonging to the normal glycine cleavage system were all down-regulated (see the cluster of one carbon units). Furthermore, two reactions, i.e. for the anaerobic nucleoside-triphosphate reductase activating system and the component ribonucleoside triphosphate reductase activase were up-regulated. These reactions needed to synthesise deoxyribonucleotides under anaerobic conditions [42,43] are showing expression patterns analogous to pyruvate formate-lyase activase [44]. Also up-regulated was the anaerobic coproporphyrinogen III oxidase. In an anaerobic environment this reaction is necessary for the biosynthesis of hemes which are essential co-factors of the electron transport chain [45].

#### Processing of hexoses

Two clusters represented the processing of hexose nutrients during anaerobic growth (Table 1). The first cluster was formed by eight up-regulated reactions (Figure 5). Connections between reactions were due to metabolites D-fructose-6-phosphate, fructose-1,6-bisphosphate and β-D-glucose-6-phosphate. Two major pathways were involved in the cluster: glycolysis, and fructose/mannose metabolism. The Embden-Meyerhof pathway is used when switching from aerobic respiration to fermentation during growth under anaerobic conditions on minimal medium with glucose [46], yielding in a strong increase of glucose consumption [29,46]. All reactions processing glucose down to fructose-1,6-biphosphate were up-regulated: Glucokinase converting glucose to glucose-6-phosphate and phosphoglucose isomerase transforming glucose-6-phosphate to fructose-6-phosphate/6-phosphofructokinase yielding fructose-1,6-bisphosphate. The increased conversion of D-fructose-6-phosphate to mannitol-1-phosphate generating the electron acceptor NAD+ normally produced in the Krebs cycle [47] explains the up-regulation of mannitol-1-phosphate 5-dehydrogenase. The higher amount of 1-phosphofructokinase is in agreement with previous findings [46]. The EIIMan transporter was up-regulated to increase the up-take of glucose. The second cluster of reactions processing hexose nutrients contained five up-regulated reactions which were connected due to the metabolites 2-keto-3-deoxy-6-phospho-gluconate, D-glyceraldehyde-3-phosphate and 1,3-diphosphateglycerate. Phosphoglycerate kinase, glyceraldehyde 3-phosphate dehydrogenase and triose phosphate isomerase are induced by anaerobiosis [48,49]. Note that they are part of the glycolytic pathway of *E. coli*. Finally, phosphogluconate dehydratase and 2-keto-3-deoxy-6-phosphogluconate aldolase which are key enzymes of the Entner-Doudoroff pathway, were up-regulated, further demonstrating anaerobic glucose metabolism [50].

#### Iron processing

Two clusters represented the processing of iron in an anaerobic environment (Table 1). The first cluster contained eight down-regulated reactions. From these, seven belonged to the complete biosynthesis pathway of enterobactin which is used by *E. coli *to scavenge iron, starting with isochorismate synthase and ending at the aryl carrier protein [51] (see Figure 6). Enterobactin biosynthesis is repressed under anaerobic conditions as it is used for aerobic iron transport [52]. Directly connected to the enterobactin pathway is enterochelin esterase. Enterochelin esterase uses enterobactin as an educt [53]. As biosynthesis of enterobactin was down-regulated, the down-regulation of enterochelin esterase is explained by the lower availability of its educts. The second cluster for iron processing contained five down-regulated reactions. Metabolites connecting the reactions were L-alanine and L-cysteine. The majority of the reactions are involved in Fe-S cluster biogenesis. The most connected node was cysteine desulfurase. This reaction assembles Fe-S complexes into Fe-S proteins to repair them when damaged during oxidative stress [54]. Under anaerobic conditions damage by oxidative stress is negligible explaining the down-regulation of cysteine desulfurase [55], whereas up-regulation as an oxidative stress response has been reported under aerobic conditions [56]. Directly linked to cysteine desulfurase was the thiamin (thiazole moiety) biosynthesis protein, which is a catalyser transferring sulfur from cysteine to the ThiS protein. It was down-regulated because during anaerobic growth a lower level of thiamin is needed compared to aerobic conditions [54]. Furthermore, selenocysteine lyase was connected to cysteine desulfurase via alanine. Selenocysteine lyase seems to be regulated by IscR and to form an alternate pathway involved in Fe-S biogenesis under aerobic conditions. In an anaerobic environment this reaction is known to be down-regulated [55].

#### Acid response

One prominent cluster was formed by 18 up-regulated reactions. Ten of these represent the complete histidine biosynthesis pathway, beginning with ATP phosphoribosyltransferase and ending at histidinal dehydrogenase (Figure 7). When growing anaerobically on glucose, *E. coli *synthesises acids via mixed acid fermentation [46,57] and histidine is used to buffer acidic milieu [58]. Another reaction in the cluster was CTP synthetase which was also up-regulated. Due to the down-regulation of NDP kinase the elevated levels of CTP synthetase are in agreement with previous findings [59] while the concrete functionality of this remains unclear. Furthermore, the cluster consisted of up-regulated reactions that needed or produced aspartate under anaerobic conditions. In yeast it was shown that the aspartate concentration is roughly 100 times higher in the cells under anaerobic conditions [60]. Generating aspartate may facilitate the biosynthesis of further amino acids and other essential compounds. *E. coli *has two known reactions catalysing the synthesis of asparagine, asparagine synthetase and aspartate-ammonia ligase. Both reactions were up-regulated during anaerobic growth, in agreement with previous findings [61]. The role of aspartate was further reinforced by the up-regulation of the GltP glutamate/aspartate DAACS transporter. Finally the cluster consisted of the starting points for the anaerobic de novo biosynthesis of NAD which were also up-regulated. This pathway uses L-aspartate to form NAD via L-aspartate oxidase and Quinolinate synthase [62,63]. Although NAD may be constitutively produced, the up-regulation of both reactions makes sense, as it has been shown that Quinolinate synthetase is inactive when exposed to oxygen [62].

#### Nucleosides metabolisms

Two clusters indicating a change in the processing of nucleosides were found (Table 1). One cluster contained eight down-regulated reactions processing GTP, GDP and dGDP. GTP cyclohydrolase I was down-regulated to limit the biosynthesis of cost intensive folate and highly abundant formate under anaerobic conditions. Similarly, GDP kinase, dGDP kinase, GDP reductase, deoxyguanylate kinase and ribonucleoside-diphosphate reductase 2 were down-regulated, which may be due to reducing the metabolism of cost intensive purines. Similarly the down-regulation of GDP diphosphokinase and deoxyguanylate kinase can be explained. The second cluster consisted of six down-regulated reactions. Edges between reactions were due to metabolites UTP, UDP, UDP-galactose, α-D-glucose 1-phosphate or dTTP. The highest connected node was UDP kinase. Interestingly, this cluster compares to the cluster above showing down-regulated processing of cost intensive nucleosides.

#### One carbon units

Six down-regulated reactions formed a cluster showing the processing of one carbon units under anaerobic conditions. Metabolites connecting the reactions were glycine, H-protein-*S*-(aminomethyldihydrolipoyl)lysine and H-protein-(lipoyl)lysine. The central reaction was glycine dehydrogenase (decarboxylating) which together with aminomethyltransferase is part of the glycine cleavage system. Although it is reported that the glycine cleavage system is active under anaerobic conditions [41], the down-regulation stems from the fact that the corresponding reaction reduces NAD^+ ^to NADH which is very costly due to the low availability of NAD^+ ^[64]. The production of one-carbon units, for which the glycine cleavage system is used [65], was taken over by glycine hydroxymethyltransferase (see switches). Furthermore, lipoyl-protein ligase A was down-regulated to reduce pyruvate dehydrogenase and to increase pyruvate formate lyase activity [66]. As a response to oxidative stress the expression of glutathione synthetase increases [67]. In an anaerobic environment no oxidative stress is prevalent, explaining the down-regulation of glutathione synthetase. Glycine-tRNA synthetase was down-regulated which may be due to reduced growth under oxygen deprivation.

#### Functional description of significant switches

64 significant switches were found (p-value ≤ 0.01, Table 2, supplement 2). The first 20 are interpreted here. Switches belonging to the same metabolic process are described in common paragraphs.

#### Formate fermentation

Five switches (1, 3, 4, 8, 15) belonged to the fermentation of formate. The following reaction-pairs were up-regulated and down-regulated respectively: formate hydrogenase complex and formyltetrahydrofolate deformylase, FocA formate FNT transporter and formyltetrahydrofolate deformylase, formate hydrogenase complex and GTP cyclohydrolase I, formate dehydrogenase and formyltetrahydrofolate deformylase, FocA formate FNT transporter and GTP cyclohydrolase I. All switches formed an intersection between degradation and formation of formate. Due to the high abundance of formate in the cell under anaerobic conditions, the formation of new formate was down-regulated (see e.g. [68]), while the degradation of formate into CO_2 _and H_2 _and the transport of formate to the periplasm was up-regulated.

#### Mixed acid fermentation and anaerobic respiration

Four switches (2, 10, 11, 14) belonged to mixed acid fermentation and anaerobic respiration. The first of these switches was formed by up-regulated acetaldehyde dehydrogenase and down-regulated ethanolamine ammonia-lyase. The reactions were connected via the metabolite acetaldehyde. *E. coli *ferments glucose via acetyl-CoA to ethanol. The first step in this fermentation is catalysed by acetaldehyde dehydrogenase converting acetyl-CoA to acetaldehyde [69]. Ethanolamine ammonia-lyase catalyses the cleavage of ethanolamine to acetaldehyde and ammonia [70]. Ethanolamine can be used as a carbon and energy source under aerobic conditions [71], resulting in a down-regulation of the reaction under anaerobic conditions. In two switches fumarate reductase was up-regulated where 5'-phosphoribosyl-4-(N-succinocarboxamide)-5-aminoimidazole lyase and adenylosuccinate lyase were down-regulated. 5'-phosphoribosyl-4-(N-succinocarboxamide)-5-aminoimidazole lyase and adenylosuccinate lyase form a bifunctional enzyme. The metabolite connecting the differently regulated reactions was fumarate in both cases. Fumarate reductase was up-regulated as it is used by *E. coli *during anaerobic growth [34] with menaquinol acting as an electron acceptor, while fumarate functions as a terminal electron donor [35]. 5'-phosphoribosyl-4-(N-succinocarboxamide)-5-aminoimidazole lyase/adenylosuccinate lyase was down-regulated to reduce the biosynthesis of purines indicating the reduced growth under oxygen deprivation. Another switch consisted of up-regulated phosphoenolpyruvate carboxylase and down-regulated aspartate transaminase, connected by the metabolite oxaloacetate. Phosphoenolpyruvate carboxylase participates in mixed-acid fermentation of glucose [72] and is therefore up-regulated during anaerobic growth. Under anaerobic conditions the citrate cycle is shortened to a reductive branch. CoA and oxaloacetate is then further processed to succinyl-coenzyme A by two possible branches, either using aspartate transaminase or malate dehydrogenase [73]. With our finding it seems that the second branch is favoured.

#### One carbon units

Two switches (6, 7) were part of the metabolism of one-carbon units. The switches consisted of up-regulated serine hydroxymethyltransferase and down-regulated reactions of the glycine cleavage system (gcv system and glycine dehydrogenase (decarboxylating)). Both reactions produce 5,10-methylene-THF and are therefore major contributors of one-carbon units in *E. coli *[65,74,75]. The switch found here indicates that under anaerobic conditions one-carbon units are more produced by serine hydroxymethyltransferase than in an aerobic environment. The glycine cleavage system reduces NAD^+ ^to NADH. This is a costly reaction as NAD^+ ^is only available in small quantities [64]. Therefore, the glycine cleavage system is down-regulated resulting in an up-regulation of serine hydroxymethyltransferase to compensate for the loss in one-carbon units.

#### Processing of hexoses

Two switches (9, 13) belonged to the processing of gluconate and glyoxylate. Both up-regulated nodes, 2-ketoaldonate reductase and 2-keto-4-hydroxyglutarate aldolase, participate in the intracellular regulation of glyoxylate levels [76,77]. Gluconokinase converts gluconate to 6-phosphogluconate which then can enter the Entner-Doudoroff or the pentose phosphate pathway [77] while 2-keto-4-hydroxyglutarate aldolase forms a part of the Entner-Doudoroff pathway. Normally, the Entner-Doudoroff pathway is used if *E. coli *grows on gluconate. It exhibits basal levels of activity of this pathway if growing on glucose [78]. This is explained by the steady production of gluconate during glucose metabolism [78]. Under anaerobic conditions on glucose, the glucose metabolism is up-regulated (see Figure 5), which is followed by increased production of gluconate and an up-regulation of gluconate processing reactions. The down-regulated reactions, glyoxylate reductase and 2-ketoaldonate reductase respectively, use NADPH as the electron donor and cooperate with gluconate reductase [76] that, under aerobic conditions, brings glyoxylate into the tricarboxylic acid cycle [76]. Under anaerobic conditions this cycle is limited, resulting in the observed down-regulation.

#### Branched chain amino acids transporters

Three switches (16, 17, 18) were formed by branched chain amino acid transporters. Up-regulated were the BrnQ branched chain amino acid LIVCS transporters (EcoCyc-ids: TRANS-RXN-126, -126B, -126A). In contrast, branched chain amino acids ABC transporters (EcoCyc-ids: ABC-15-RXN, ABC-35-RXN, ABC-36-RXN) were down-regulated. The ABC transporters need costly ATP [79] resulting in a down-regulation under anaerobic conditions. To compensate for the loss of the high affinity ATP using transporters the low affinity branched chain amino acid LIVCS transporters were up-regulated [80].

#### Miscellaneous

An unexpected contrarily regulated reaction pair (switch 20) was formed by up-regulated phosphatidylglycerophosphate synthase and down-regulated CDP-diacylglycerol pyrophosphatase. *E. coli *uses phosphatidylglycerophosphate synthase to catalyse the biosynthesis of acidic phospholipids [81] synthesising phosphatidylglycerol. It plays a major role in translocation of e.g. trimethylamine N-oxide reductase [82], a reaction used for anaerobic respiration [83]. Unfortunately, not much functional knowledge exits about the down-regulated CDP-diacylglycerol pyrophosphatase. Switch 12 consisted of up-regulated CTP synthetase and down-regulated UDP-glucose-hexose-1-phosphate uridylyltransferase. The up-regulation of CTP-synthetase agrees to previous findings [59] although the reason for this remains to be investigated further. Two switches (5, 19) were inexplicable to us (see Conclusions).

#### Comparison to a standard method

We compared the list of genes extracted with our technique to a standard t-test which did not take any network information into account. A mutant-corrected t-test (see Methods) was run on the gene expression levels for the corresponding reactions. Table 3 shows the results for the first 40 highest ranking features. All except six reactions were also found by our technique. The top three reactions (1, 2, 3 of Table 3) are involved in fermentation of formate and were also found with our technique (Table 1, Figure 4). Our technique was capable detecting whole pathways that occurred in the list of our top ranking features. However, the standard method did not detect such pathways or sub-graphs (discussed in the text, see above) supporting our concept for identifying functionally relevant sub-graphs. The six reactions 10, 13, 15, 25, 29, 32 were not extracted by our technique. Five of these reactions were not found due to the network construction: Unspecific metabolites were deleted resulting in the deletion of reactions that catalyse unspecific substrates, such as pyruvate kinase, glutamate dehydrogenase (NADP+), NAD kinase, NADH oxidoreductase and RhtB homoserine Rht transporter. Putative reactions with undefined metabolites like N-acetyl-anhydromuramyl-L-alanine-amidase, were also not included into the studied metabolic network and could therefore not be identified.

**Table 3:** Discriminative reactions from a t-test. .

**Rank**	**Reaction**	**Regulation**	**P-value**	**Found also with our technique**
1	formate hydrogenlyase complex	1	2.99E-19	Yes
2	FocA formate FNT transporter	1	3.18E-16	Yes
3	pyruvate formate-lyase	1	7.29E-15	Yes
4	aminomethyltransferase	-1	3.33E-14	Yes
5	gcv system	-1	1.67E-12	Yes
6	3-methyl-2-oxobutanoate hydroxymethyltransferase	1	3.92E-12	Yes
7	glycine dehydrogenase (decarboxylating)	-1	5.52E-12	Yes
8	PFL-deactivase	1	2.01E-11	Yes
9	acetaldehyde dehydrogenase	1	2.01E-11	Yes
10	pyruvate kinase	1	2.55E-11	No
11	fumarate reductase	1	2.69E-11	Yes
12	enolase	1	2.87E-11	Yes
13	N-acetylmuramyl-L-alanine amidase	1	3.10E-11	No
14	formate dehydrogenase	1	3.30E-11	Yes
15	glutamate dehydrogenase (NADP+)	1	4.21E-11	No
16	mannonate dehydratase	-1	7.97E-11	Yes
17	pyruvate formate-lyase activating enzyme	1	1.91E-10	Yes
18	pyruvate formate-lyase activating enzyme	1	1.91E-10	Yes
19	triose phosphate isomerase	1	2.25E-10	Yes
20	glutamyl-tRNA reductase	1	3.21E-10	Yes
21	histidine-phosphate aminotransferase	1	3.35E-10	Yes
22	2-keto-4-hydroxyglutarate aldolase	1	7.98E-10	Yes
23	2-keto-3-deoxy-6-phosphogluconate aldolase	1	7.98E-10	Yes
24	oxaloacetate decarboxylase	1	7.98E-10	Yes
25	putative NAD+ kinase	1	1.29E-09	No
26	6-phosphofructokinase-1	1	1.29E-09	Yes
27	mannose-6-phosphate isomerase	1	1.37E-09	Yes
28	Outer Membrane Ferrichrome Transport System	-1	1.57E-09	Yes
29	NADH oxidoreductase	1	2.19E-09	No
30	isocitrate dehydrogenase kinase	-1	4.61E-09	Yes
31	isocitrate dehydrogenase phosphatase	-1	4.61E-09	Yes
32	RhtB homoserine Rht Transporter	1	5.70E-09	No
33	histidinol-phosphatase	1	7.39E-09	Yes
34	imidazoleglycerol-phosphate dehydratase	1	7.39E-09	Yes
35	Outer Membrane Ferric Enterobactin Transport System	-1	1.49E-08	Yes
36	phosphoenolpyruvate carboxylase	1	2.38E-08	Yes
37	tetrahydrodipicolinate succinylase	1	2.90E-08	Yes
38	imidazole glycerol phosphate synthase	1	3.36E-08	Yes
39	3-hydroxy acid dehydrogenase	1	3.59E-08	Yes
40	branched chain amino acids ABC transporter	-1	4.83E-08	Yes

The 40 first ranking reactions when applying a mutant and multiple testing corrected t-test directly without any network information. Shaded rows were found only by this standard method (see text)

## Conclusion

We applied simplified first- and second-order Haar-wavelet-transformations to select combined transcription levels of reaction-pairs. We chose the Haar wavelets as they enable connecting two discrete data points (reaction pairs in our case) in a straightforward way. Furthermore, we searched for common *and *opposing responses between combined gene expression data which matched well to the shape of the Haar wavelet filters. Through using this approach we gained substantial insight into the metabolic regulation of *E. coli *upon the transition from oxygen-rich to oxygen-deprived conditions. Such an approach complements to the original idea of DeRisi and co-workers to use microarray technology for discovering system changes. For example, they revealed changes in yeast metabolism during the diauxic shift [84]. In the study presented here, we discovered a broad spectrum of responses including direct responses to limited oxygen and changing buffer conditions. As a response to limited oxygen, we identified an up-regulation in glycolysis, other hexose metabolisms, mixed acid fermentation, formate fermentation and the metabolism of aspartate. In summary, we see two interesting implications for our study, (i) data analysis: the implementation of the Haar-wavelet technique on small pairs of nodes is well suited for revealing significant patterns in a cellular network; and (ii) functional: many pathways are regulated on a transcriptional level supporting the concept of hierarchical control analysis for micro-organisms [85,86].

The formate fermentation showed an interesting switch like behaviour: for oxygen deprived conditions the degradation of formate was up-regulated while its cost-intensive production was down-regulated. Note that this may be more difficult to reveal when using smoothing techniques (as e.g. [17,18]) and demonstrates the benefit of using wavelets. Furthermore, a decrease in the metabolism of iron was detected as a response to reduced oxygen availability. Interestingly, this agrees with Faith *et al*. who analysed a large compendium of 445 microarrays for *E. coli *including a variety of different oxygen conditions [9]. They showed that PdHR which regulates the central metabolism, is also involved in regulating the fec operon which encodes genes for iron transport. We discovered that the entire histidine biosynthesis pathway was up-regulated as a possible response to accumulation of acid products in batch culture [58]. However, essential sub-graphs were not only detected in an isolated form, but also in relation to connected pathways which depended on the same metabolites. E.g., the cluster containing the histidine biosynthesis pathway (Figure 7) also contained components for metabolism of aspartate and glutamine. In addition, the cluster of formate fermentation (Figure 4) included parts of the aspartate metabolism. This reflects the unspecific hub-like nature of key metabolites such as L-glutamine and aspartate connecting several pathways. Significant switches supported the yielded adaptation mechanisms of *E. coli *to changing oxygen abundance, as e.g., switches pertaining to the fermentation of formate and mixed acids.

In our previous study, we used the same microarray dataset and extracted discriminate patterns of highly connected regions in the network [26]. In comparison to the present study here, we got a good consistency of the extracted pathways (glycolysis, aspartate metabolism, formate fermentation, pyruvate metabolism). In the study presented here, we elucidated some new pathways, i.e. the histidine biosynthesis, enterobactin biosynthesis (oxidative stress response), the aerobic part of the TCA cycle, and hexoses and one-carbon-units processing. It is of note that histidine biosynthesis and the biosynthesis of enterobactin are linear chains in the network. In contrast to the previous method, such linear chains can be well recognised by the method we present here which couples pairs of nodes. However, the previous method recognised two interesting, highly-connected regions which were not indicated using our new method (the interface between glycolysis and NAD biosynthesis, and the biosynthesis of lysine, see [26]). Two observed switches remain explainable. One switch (switch 19) consisted of 3-hydroxy acid dehydrogenase and phosphoserine phosphatase. In the second case (switch 5) up-regulated 3-methyl-2-oxobutanoate hydroxymethyltransferase and down-regulated 2-dehydropantoate reductase directly followed the up-regulated reaction in the biosynthesis of pantothenate. These results may reveal an incomplete understanding of these metabolic components and the need for further experimental investigation.

On simulated data, the accuracy and precision was significantly better in comparison to the standard method. This allowed us to use a p-value of 0.01 and to focus on more significant changes. We compared our technique with a standard method extracting lists of discriminative genes from the expression data without taking gene relationships into account. We were able to detect all relevant reactions that could also be found by the standard method. In contrast, the standard method failed to reveal comprehensive functional pathways. However, for future studies a general method to validate the functionality of such a broad spectrum of newly revealed pathways remains to be developed. Nevertheless, our technique might be used for analysing signalling networks, e.g., to identify discriminative regulations in cancers with different prognosis, even though reaction and signalling levels might be less related to gene expression levels for higher organisms. Further methodological advances might also include the addition of protein post-transcriptional regulation and the application of more complex image processing methods.

## Methods

### Establishing the metabolic network

All metabolic reactions were extracted from the EcoCyc database (Version 10.0) [87]. A graph was established by defining neighbours of reactions: Two reactions were neighbours if a metabolite existed that was the product of one reaction and the substrate for the other. In this representation the nodes of the graph were the reactions while edges were defined by the metabolites. Metabolites were discarded that were highly connected and therefore pathway unspecific, such as water, oxygen, major coenzymes and prostethic groups. This approach resulted in a graph with 1196 nodes and 3650 edges.

### Mapping gene expression data onto nodes of the network

Raw intensity values of gene expression data were collected from the work of Covert et al. in which mRNA levels of all open reading frames of *E. coli *using Affymetrix oligo microarrays were determined [28]. The data was downloaded from the ASAP database [88] and normalised with the variance normalisation method [89]. 43 hybridisations of the following samples were selected: strain K-12 MG 1655, wild-type, *ΔarcA, ΔappY, Δfnr, ΔoxyR, ΔsoxS *single mutants and the *ΔarcAΔfnr *double mutant. The mutated genes are key transcriptional regulators of the oxygen response [28]. They effect a major portion of all genes in *E. coli*. All gene expression experiments were done in triplicate under aerobic and anaerobic conditions, except for anaerobic wild-type which was repeated four times. The gene expression data of each data-set was mapped onto the corresponding reactions of the transcribed proteins. If a reaction was catalysed by a complex of proteins the minimal expression value of the genes involved was taken as the value of the corresponding complex. The expression data of all samples was mapped onto each network, yielding 43 different patterns for each graph.

### Generating feature modules

To discover specific expression patterns and textures in the network we calculated features with the Haar-wavelet transformation consisting of gene expression combinations of neighbouring reaction-pairs. Haar-wavelet transformations add and subtract the values of neighbouring pairs of nodes and multiply them with a constant factor: Be r_0_, r_1 _the gene expression values of a pair of reactions, respectively. Applying the transform yields the feature modules f_0_, f_1_:

(f1f0)=12(111−1)(r0r1).
 MathType@MTEF@5@5@+=feaafiart1ev1aaatCvAUfKttLearuWrP9MDH5MBPbIqV92AaeXatLxBI9gBaebbnrfifHhDYfgasaacH8akY=wiFfYdH8Gipec8Eeeu0xXdbba9frFj0=OqFfea0dXdd9vqai=hGuQ8kuc9pgc9s8qqaq=dirpe0xb9q8qiLsFr0=vr0=vr0dc8meaabaqaciaacaGaaeqabaqabeGadaaakeaadaqadaqaauaabeqaceaaaeaacqWGMbGzdaWgaaWcbaGaeGymaedabeaaaOqaaiabdAgaMnaaBaaaleaacqaIWaamaeqaaaaaaOGaayjkaiaawMcaaiabg2da9maalaaabaGaeGymaedabaWaaOaaaeaacqaIYaGmaSqabaaaaOWaaeWaaeaafaqabeGacaaabaGaeGymaedabaGaeGymaedabaGaeGymaedabaGaeyOeI0IaeGymaedaaaGaayjkaiaawMcaamaabmaabaqbaeqabiqaaaqaaiabdkhaYnaaBaaaleaacqaIWaamaeqaaaGcbaGaemOCai3aaSbaaSqaaiabigdaXaqabaaaaaGccaGLOaGaayzkaaGaeiOla4caaa@4437@

See also [90] for more details. The Haar wavelet transform can be regarded as a low pass filter when performing the summation and a high pass filter when calculating the difference between neighbouring value pairs. Both filters were applied on all pairs of nodes connected by an edge resulting in calculated feature modules.

### Statistical testing of the feature modules

All Haar-wavelet generated features were tested by a multiple t-test between aerobic and anaerobic conditions. To correct for potential influences coming from individual mutants, t-tests were performed for every constellation of samples excluding the sample of one particular mutant, respectively. The wild type sample was never excluded. From this outcome the worst (highest) p-value for each feature was selected. All p-values were corrected for multiple testing (Bonferroni, see [27]). Features were then ranked according to their p-value.

### Clustering of significant reaction pairs

All significant feature modules were extracted (p-value ≤ 0.01). Sub-graphs were put up by connecting the found significant feature modules (reaction-pairs). This resulted in five larger sub-graphs. To facilitate the interpretation of the found sub-graphs, nodes with equal expression behaviour (up-, down-regulation) were grouped together. To reduce random fluctuations we focused only on larger patterns, i.e. clusters with a cluster size smaller than five were discarded. In total 10 clusters were extracted. Reaction-pairs having one up- and one down-regulated node were regarded as switches. They were extracted if their p-value was below 0.01.

Note, that our method yielding these clusters is based on two steps: generating feature modules and combining those with a common response yielding significant clusters of co-expression. The first step compares to a low and a high pass filter of the first order Haar-wavelet-transformation, respectively. The second step compares to a low pass filter of the second order Haar-wavelet-transform.

### Analysing the found clusters and switches in-depth

All extracted clusters and switches were functionally characterised (see Results and Discussion). An in-depth analysis was performed by scanning the literature. Finally, the analysed clusters and switches were assembled yielding an overall map of the metabolic changes.

## Authors' contributions

GS and RK conceptualised and designed the method. GS analysed the data. RK, GS and MZ analysed and interpreted the data on its biological content. The manuscript was written by GS, MZ and RK. RE revised it critically. All authors read and approved the final manuscript.

## Supplementary Material

Additional file 1 Supplement 1: Extracted network clusters with EcoCyc identifiers. The extracted network clusters with the identifiers from the EcoCyc database. Click here for file

Additional file 2 Supplement 2: All significant switches in the network. List of all significant switches found in the network. Click here for file
