# Supplementary material for: Using gene expression data and network topology to detect substantial pathways, clusters and switches during oxygen deprivation of Escherichia coli
Source: BMC Bioinformatics. 2007 May 8;8:149. doi: 10.1186/1471-2105-8-149 (PMC1884177; doi:10.1186/1471-2105-8-149)
Supplement: Additional file 1 — Supplement 1: Extracted network clusters with EcoCyc identifiers. The extracted network clusters with the identifiers from the EcoCyc database. [file 1471-2105-8-149-S1.doc]

**Supplement 1: Extracted network clusters with EcoCyc identifiers**

| **Processing of pyruvate , fermentation of formate, anaerobic respiration, synthesis of deoxyribonucleosides** | |
| --- | --- |
| **EcoCyc-id** | **Reaction** |
| **1st cluster** | |
| ARGSUCCINLYA-RXN | argininosuccinate lyase |
| ASPARTASE-RXN | aspartate ammonia-lyase |
| DIMESULFREDUCT-RXN | dimethyl sulfoxide reductase |
| DIOHBUTANONEPSYN-RXN | 3,4-dihydroxy-2-butanone 4-phosphate synthase |
| FHLMULTI-RXN | formate hydrogenlyase complex |
| FORMATEDEHYDROG-RXN | formate dehydrogenase |
| PYRUVFORMLY-RXN | pyruvate formate-lyase |
| R601-RXN | fumarate reductase |
| TRANS-RXN-1 | FocA formate FNT transporter |
| **2nd cluster** | |
| 1.97.1.4-A-RXN | pyruvate formate-lyase activating enzyme |
| HEMN-RXN | coproporphyrinogen III oxidase, anaerobic |
| NRDACTMULTI-RXN | anaerobic nucleoside-triphosphate reductase activating system |
| PFLDEACTIV-RXN | PFL-deactivase |
| RNTRACTIV-RXN | ribonucleoside triphosphate reductase activase |
| RXN0-949 | lipoate synthase |
| TDCEACT1-RXN | pyruvate formate-lyase activating enzyme |
| **Processing of C6-nutrients** | |
| **EcoCyc-id** | **Reaction** |
| **1st cluster** | |
| 1PFRUCTPHOSN-RXN | 1-phosphofructokinase |
| 6PFRUCTPHOS-RXN | 6-phosphofructokinase |
| 6-PHOSPHO-BETA-GLUCOSIDASE-RXN | 6-phospho-β-glucosidase |
| GLUCOKIN-RXN | glucokinase |
| MANNPDEHYDROG-RXN | mannitol-1-phosphate 5-dehydrogenase |
| MANNPISOM-RXN | mannose-6-phosphate isomerase |
| PGLUCISOM-RXN | phosphoglucose isomerase |
| TRANS-RXN-158A | EIIMan |
| **2nd cluster** | |
| GAPOXNPHOSPHN-RXN | glyceraldehyde 3-phosphate dehydrogenase |
| KDPGALDOL-RXN | 2-keto-3-deoxy-6-phosphogluconate aldolase |
| PGLUCONDEHYDRAT-RXN | phosphogluconate dehydratase |
| PHOSGLYPHOS-RXN | phosphoglycerate kinase |
| TRIOSEPISOMERIZATION-RXN | triose phosphate isomerase |

| **Processing of iron** | |
| --- | --- |
| **EcoCyc-id** | **Reaction** |
| **1st cluster** | |
| DHBAMPLIG-RXN | 2,3-dihydroxybenzoate-AMP ligase |
| DHBDEHYD-RXN | 2,3-dihydro-2,3-dihydroxybenzoate dehydrogenase |
| ENTF-RXN | serine activating enzyme |
| ENTG-RXN | aryl carrier protein |
| ENTMULTI-RXN | enterobactin synthase multienzyme complex |
| ISOCHORMAT-RXN | isochorismatase |
| ISOCHORSYN-RXN | isochorismate synthase |
| RXN0-1661 | enterochelin esterase |
| **2nd cluster** | |
| RXN0-308 | cysteine desulfurase |
| SELENOCYSTEINE-LYASE-RXN | selenocysteine lyase |
| THIFIS-RXN | thiamin (thiazole moiety) biosynthesis protein |
| TRANS-RXN-125 | YaaJ alanine AGSS transporter |
| VALINE-PYRUVATE-AMINOTRANSFER-RXN | valine-pyruvate aminotransferase |
| **Acid response** | |
| **EcoCyc-id** | **Reaction** |
| ASNSYNA-RXN | aspartate-ammonia ligase |
| ASNSYNB-RXN | asparagine synthetase B |
| ATPPHOSPHORIBOSYLTRANS-RXN | ATP phosphoribosyltransferase |
| CDPDIGLYSYN-RXN | CDP-diglyceride synthetase |
| CTPSYN-RXN | CTP synthetase |
| GLUTAMIDOTRANS-RXN | imidazole glycerol phosphate synthase |
| HISTALDEHYD-RXN | histidinal dehydrogenase |
| HISTAMINOTRANS-RXN | histidinol-phosphate aminotransferase |
| HISTCYCLOHYD-RXN | phosphoribosyl-AMP cyclohydrolase |
| HISTIDPHOS-RXN | histidinol-phosphatase |
| HISTOLDEHYD-RXN | histidinol dehydrogenase |
| HISTPRATPHYD-RXN | phosphoribosyl-ATP pyrophosphatase |
| IMIDPHOSDEHYD-RXN | imidazoleglycerol-phosphate dehydratase |
| L-ASPARTATE-OXID-RXN | L-aspartate oxidase |
| PRIBFAICARPISOM-RXN | phosphoribosylformimino-5-amino-1-phosphoribosyl-4-imidazole carboxamide isomerase |
| QUINOLINATE-SYNTHE-MULTI-RXN | quinolinate synthase complex |
| RXN0-1241 | protein-(glutamine-N5) methyltransferase |
| TRANS-RXN-122A | aspartate DAACS transporter |

| **Processing of tri-phosphates** | |
| --- | --- |
| **EcoCyc-id** | **Reaction** |
| **1st cluster** | |
| DGDPKIN-RXN | dGDP kinase |
| GDPKIN-RXN | nucleoside diphosphate kinase |
| GDPREDUCT-RXN | ribonucleoside-diphosphate reductase |
| GMKALT-RXN | deoxyguanylate kinase |
| GTP-CYCLOHYDRO-I-RXN | GTP cyclohydrolase I |
| GUANYL-KIN-RXN | guanylate kinase |
| PPGPPSYN-RXN | guanosine-3',5'-bis(diphosphate) 3'-diphosphatase |
| RXN0-748 | ribonucleoside-diphosphate reductase |
| **2nd cluster** | |
| DTDPGLUCOSEPP-RXN | dTDP-glucose pyrophosphorylase |
| DTDPKIN-RXN | dTDP kinase |
| GALACTURIDYLYLTRANS-RXN | UDP-glucose-hexose-1-phosphate uridylyltransferase |
| GALPMUT-RXN | UDP-galactopyranose mutase |
| UDPKIN-RXN | nucleoside diphosphate kinase |
| UTPHEXPURIDYLYLTRANS-RXN | galactose-1-phosphate uridylyltransferase |
| **Processing of one carbon units** | |
| **EcoCyc-id** | **Reaction** |
| GCVMULTI-RXN | gcv system |
| GCVP-RXN | glycine dehydrogenase (decarboxylating) |
| GCVT-RXN | aminomethyltransferase |
| GLUTATHIONE-SYN-RXN | glutathione synthetase |
| GLYCINE--TRNA-LIGASE-RXN | glycyl-tRNA synthetase |
| RXN0-1141 | lipoyl-protein ligase A |
