# Supplementary material for: Using gene expression data and network topology to detect substantial pathways, clusters and switches during oxygen deprivation of Escherichia coli
Source: BMC Bioinformatics. 2007 May 8;8:149. doi: 10.1186/1471-2105-8-149 (PMC1884177; doi:10.1186/1471-2105-8-149)
Supplement: Additional file 2 — Supplement 2: All significant switches in the network. List of all significant switches found in the network. [file 1471-2105-8-149-S2.doc]

**Supplement 2: All extracted switches in the network**

|  | **Up-regulated reaction** | |  | **Down-regulated reaction** | |  |
| --- | --- | --- | --- | --- | --- | --- |
| **Rank** | **EcoCyc-ids** | **Reactions** | **Metabolites** | **EcoCyc-ids** | **Reactions** | **p-value** |
| 1 | FHLMULTI-RXN | formate hydrogenlyase complex | formate | FORMYLTHFDEFORMYL-RXN | formyltetrahydrofolate deformylase | 4.67E-14 |
| 2 | ACETALD-DEHYDROG-RXN | acetaldehyde dehydrogenase | acetaldehyde | ETHAMLY-RXN | ethanolamine ammonia-lyase | 1.25E-12 |
| 3 | TRANS-RXN-1 | FocA formate FNT transporter | formate | FORMYLTHFDEFORMYL-RXN | formyltetrahydrofolate deformylase | 5.81E-12 |
| 4 | FHLMULTI-RXN | formate hydrogenlyase complex | formate | GTP-CYCLOHYDRO-I-RXN | GTP cyclohydrolase I | 1.65E-11 |
| 5 | 3-CH3-2-OXOBUTANOATE-OH-CH3-XFER-RXN | 3-methyl-2-oxobutanoate hydroxymethyltransferase | 2-dehydropantoate | 2-DEHYDROPANTOATE-REDUCT-RXN | 2-dehydropantoate reductase | 2.33E-11 |
| 6 | GLYOHMETRANS-RXN | serine hydroxymethyltransferase | tetrahydrofolate, 5,10-methylene-THF, glycine | GCVMULTI-RXN | gcv system | 6.69E-09 |
| 7 | GLYOHMETRANS-RXN | serine hydroxymethyltransferase | glycine | GCVP-RXN | glycine dehydrogenase (decarboxylating) | 2.07E-08 |
| 8 | FORMATEDEHYDROG-RXN | formate dehydrogenase | formate | FORMYLTHFDEFORMYL-RXN | formyltetrahydrofolate deformylase | 1.30E-07 |
| 9 | 4OH2OXOGLUTARALDOL-RXN | 2-keto-4-hydroxyglutarate aldolase | glyoxylate | GLYOXYLATE-REDUCTASE-(NADP+)-RXN | glyoxylate reductase B,glyoxylate reductase | 1.74E-07 |
| 10 | R601-RXN | fumarate reductase | fumarate | AICARSYN-RXN | 5'-phosphoribosyl-4-(N-succinocarboxamide)-5-aminoimidazole lyase | 2.00E-07 |
| 11 | R601-RXN | fumarate reductase | fumarate | AMPSYN-RXN | adenylosuccinate lyase | 2.00E-07 |
| 12 | CTPSYN-RXN | CTP synthetase | UTP | UTPHEXPURIDYLYLTRANS-RXN | galactose-1-phosphate uridylyltransferase | 3.16E-07 |
| 13 | GLUCONOKIN-RXN | gluconokinase | gluconate | 1.1.1.215-RXN | 2-ketoaldonate reductase | 1.36E-05 |
| 14 | PEPCARBOX-RXN | phosphoenolpyruvate carboxylase | oxaloacetate | ASPAMINOTRANS-RXN | aspartate transaminase | 2.00E-05 |
| 15 | TRANS-RXN-1 | FocA formate FNT transporter | formate | GTP-CYCLOHYDRO-I-RXN | GTP cyclohydrolase I | 3.35E-05 |
| 16 | TRANS-RXN-126 | BrnQ branched chain amino acid LIVCS transporters | L-isoleucine | ABC-15-RXN | branched chain amino acids ABC transporters | 3.78E-05 |
| 17 | TRANS-RXN-126B | BrnQ branched chain amino acid LIVCS transporters | L-leucine | ABC-35-RXN | branched chain amino acids ABC transporters | 3.78E-05 |
| 18 | TRANS-RXN-126A | BrnQ branched chain amino acid LIVCS transporters | L-valine | ABC-36-RXN | branched chain amino acids ABC transporters | 3.78E-05 |
| 19 | RXN0-2201 | 3-hydroxy acid dehydrogenase | L-serine | PSERPHOSPHA-RXN | phosphoserine phosphatase | 4.33E-05 |
| 20 | PHOSPHAGLYPSYN-RXN | phosphatidylglycerophosphate synthase | a CDP-diacylglycerol, CMP | CDPDIGLYPYPHOSPHA-RXN | CDP-diacylglycerol pyrophosphatase | 4.78E-05 |
| 21 | CTPSYN-RXN | CTP synthetase | CTP | CDPKIN-RXN | CDP kinase | 5.42E-05 |
| 22 | CTPSYN-RXN | CTP synthetase | UTP | UDPKIN-RXN | UDP kinase | 5.42E-05 |
| 23 | ASNSYNB-RXN | asparagine synthetase B | L-aspartate, L-glutamate | ASPAMINOTRANS-RXN | aspartate transaminase | 5.81E-05 |
| 24 | FORMATEDEHYDROG-RXN | formate dehydrogenase | formate | GTP-CYCLOHYDRO-I-RXN | GTP cyclohydrolase I | 6.91E-05 |
| 25 | PHOSPHAGLYPSYN-RXN | phosphatidylglycerophosphate synthase | CMP | RXN0-302 | 2-C-methyl-D-erythritol 2,4-cyclodiphosphate synthase | 8.92E-05 |
| 26 | RXN0-1021 | pH 2.5 acid phosphatase,GTPase | GDP | PPGPPSYN-RXN | guanosine-3',5'-bis(diphosphate) 3'-diphosphatase | 9.68E-05 |
| 27 | ACETOACETYL-COA-TRANSFER-RXN | acetoacetyl-CoA transferase | acetate | CITC-RXN | citrate lyase ligase | 0.00016745 |
| 28 | CDPDIGLYSYN-RXN | CDP-diglyceride synthetase | CTP | CDPKIN-RXN | CDP kinase | 0.00019794 |
| 29 | DCTP-DEAM-RXN | dCTP deaminase | dCTP | DCDPKIN-RXN | dCDP kinase | 0.00020212 |
| 30 | NACGLCTRANS-RXN | N-acetylglucosaminyl transferase | UDP | UDPKIN-RXN | UDP kinase | 0.00028613 |
| 31 | DTMPKI-RXN | dTMP kinase | dTDP | DTDPKIN-RXN | dTDP kinase | 0.00028654 |
| 32 | CDPDIGLYSYN-RXN | CDP-diglyceride synthetase | a CDP-diacylglycerol, an L-phosphatidate | CDPDIGLYPYPHOSPHA-RXN | CDP-diacylglycerol pyrophosphatase | 0.00034317 |
| 33 | RXN0-1021 | pH 2.5 acid phosphatase,GTPase | GDP,GTP | GDPKIN-RXN | GDP kinase | 0.00035984 |
| 34 | GLYOXI-RXN | glyoxalase I | glutathione | GSHTRAN-RXN | glutathione transferase | 0.00040215 |
| 35 | GLYOXI-RXN | glyoxalase I | glutathione | GST-RXN | glutathione transferase | 0.00040215 |
| 36 | ORNCARBAMTRANSFER-RXN | ornithine carbamoyltransferase,ornithine carbamoyltransferase | L-ornithine | ACETYLORNDEACET-RXN | acetylornithine deacetylase | 0.00065877 |
| 37 | RXN0-723 | ribonucleoside-triphosphate reductase | CTP | CDPKIN-RXN | CDP kinase | 0.0009103 |
| 38 | RXN0-746 | ribonucleoside-triphosphate reductase | GTP | GDPKIN-RXN | GDP kinase | 0.0009103 |
| 39 | RIBONUCLEOSIDE-TRIP-REDUCT-RXN | ribonucleoside-triphosphate reductase | Ribonucleoside-Triphosphates | NUCLEOSIDE-DIP-KIN-RXN | nucleoside diphosphate kinase | 0.0009103 |
| 40 | RXN0-724 | ribonucleoside-triphosphate reductase | UTP | UDPKIN-RXN | UDP kinase | 0.0009103 |
| 41 | UDPACYLGLCNACDEACETYL-RXN | UDP-3-O-acyl-N-acetylglucosamine deacetylase | acetate | CITC-RXN | citrate lyase ligase | 0.00202052 |
| 42 | ALANINE--TRNA-LIGASE-RXN | alanyl-tRNA synthetase | L-alanine | RXN0-308 | cysteine desulfurase | 0.00228665 |
| 43 | CATAL-RXN | hydroperoxidase I,hydroperoxidase II | H_2_O_2_ | RXN0-267 | thiol peroxidase 2,thiol peroxidase | 0.00251736 |
| 44 | GLYOHMETRANS-RXN | serine hydroxymethyltransferase | L-serine | PSERPHOSPHA-RXN | phosphoserine phosphatase | 0.00272314 |
| 45 | 4OH2OXOGLUTARALDOL-RXN | 2-keto-4-hydroxyglutarate aldolase | glyoxylate | ISOCIT-CLEAV-RXN | isocitrate lyase | 0.0031454 |
| 46 | RXN0-724 | ribonucleoside-triphosphate reductase | UTP | UTPHEXPURIDYLYLTRANS-RXN | galactose-1-phosphate uridylyltransferase | 0.00326514 |
| 47 | RXN0-1021 | pH 2.5 acid phosphatase,GTPase | GDP | RXN0-748 | ribonucleoside-diphosphate reductase 2 | 0.00353104 |
| 48 | 3-CH3-2-OXOBUTANOATE-OH-CH3-XFER-RXN | 3-methyl-2-oxobutanoate hydroxymethyltransferase | 2-keto-isovalerate | VALINE-PYRUVATE-AMINOTRANSFER-RXN | valine-pyruvate aminotransferase | 0.00362304 |
| 49 | RXN0-2161 | seryl-tRNA synthetase | L-serine, diphosphate | ENTF-RXN | serine activating enzyme | 0.00366558 |
| 50 | SERINE--TRNA-LIGASE-RXN | seryl-tRNA synthetase | L-serine, diphosphate | ENTF-RXN | serine activating enzyme | 0.00366558 |
| 51 | RXN0-2161 | seryl-tRNA synthetase | L-serine | PSERPHOSPHA-RXN | phosphoserine phosphatase | 0.00391728 |
| 52 | SERINE--TRNA-LIGASE-RXN | seryl-tRNA synthetase | L-serine | PSERPHOSPHA-RXN | phosphoserine phosphatase | 0.00391728 |
| 53 | ACETYLORNTRANSAM-RXN | acetylornithine transaminase | N-acetyl-L-ornithine, acetate | ACETYLORNDEACET-RXN | acetylornithine deacetylase | 0.00403334 |
| 54 | 4OH2OXOGLUTARALDOL-RXN | 2-keto-4-hydroxyglutarate aldolase | glyoxylate | MALSYN-RXN | malate synthase G,malate synthase A | 0.00431505 |
| 55 | TRANS-RXN-142 | Mtr tryptophan ArAAP transporter | indole | RXN0-2382 | tryptophan synthase, β subunit dimer | 0.0048058 |
| 56 | CATAL-RXN | hydroperoxidase I,hydroperoxidase II | H_2_O_2_ | RXN0-1461 | coproporphyrinogen oxidase, aerobic | 0.00510559 |
| 57 | RXN0-2201 | 3-hydroxy acid dehydrogenase | L-serine | RXN0-2382 | tryptophan synthase, β subunit dimer | 0.00520985 |
| 58 | RXN0-279 | cysteine sulfinate desulfinase | L-alanine | RXN0-308 | cysteine desulfurase | 0.00561034 |
| 59 | R601-RXN | fumarate reductase | fumarate | FUMHYDR-RXN | fumarase A,fumarase B,fumarase C | 0.00594138 |
| 60 | SAICARSYN-RXN | phosphoribosylaminoimidazole-succinocarboxamide synthase | L-aspartate | ASPARAGHYD-RXN | asparaginase III,asparaginase I,asparaginase II | 0.00623828 |
| 61 | ACETALD-DEHYDROG-RXN | acetaldehyde dehydrogenase | NAD, acetaldehyde, NADH | ALCOHOL-DEHYDROG-RXN | ethanol dehydrogenase,alcohol dehydrogenase | 0.00711119 |
| 62 | TRANS-RXN-122A | GltP glutamate/aspartate DAACS transporter | L-aspartate | ASPARAGHYD-RXN | asparaginase III,asparaginase I,asparaginase II | 0.00826278 |
| 63 | RXN0-2201 | 3-hydroxy acid dehydrogenase | L-serine | ENTF-RXN | serine activating enzyme | 0.00875425 |
| 64 | SAICARSYN-RXN | phosphoribosylaminoimidazole-succinocarboxamide synthase | L-aspartate | ASPAMINOTRANS-RXN | aspartate transaminase | 0.00949873 |
